# Low-Cycle Fatigue Behavior of the Novel Steel and 30SiMn2MoV Steel at 700 °C

**DOI:** 10.3390/ma13245753

**Published:** 2020-12-16

**Authors:** Chao Zhao, Jin Zhang, Jiawei Fu, Yong Lian, Zunjun Zhang, Cheng Zhang, Jinfeng Huang

**Affiliations:** 1 Institute for Advanced Materials and Technology, University of Science and Technology Beijing, Beijing 100083, China; zhaoc39@163.com (C.Z.); zhangjin@ustb.edu.cn (J.Z.); liany09@126.com (Y.L.); 2 Department of Mechanical Engineering, Nanjing University of Science and Technology, Nanjing 210094, China; jwfu@njust.edu.cn; 3 State Key Laboratory for Advanced Metals and Materials, University of Science and Technology Beijing, Beijing 100083, China; zzjzzj555@163.com

**Keywords:** the novel steel, 30SiMn2MoV steel, high temperature LCF, strain energy density, fatigue fracture

## Abstract

As a newly developed gun barrel steel, the novel steel has shown excellent high-temperature strength, high resistance to wear and erosion, contributing to the superior ballistic life of gun barrels. As ballistic life increases, the fatigue life becomes essential for the safety and reliability of gun barrels. This paper presents a comparison of the low cycle fatigue (LCF) behaviors between a novel steel and 30SiMn2MoV steel at 700 °C. A strain-controlled fatigue test was carried out on the novel steel and 30SiMn2MoV steel in the strain range from 0.2 to 0.6%. The cyclic stress response behaviors of the novel steel and 30SiMn2MoV steel show cyclic softening behavior. In addition, the shape of the hysteresis rings of the novel steel and 30SiMn2MoV steel exhibit no-Masing model behavior. Energy–life relationships results show that the novel steel has higher fatigue resistance than the 30SiMn2MoV steel at 700 °C. The results of fatigue fracture analysis show that the failure mode of the 30SiMn2MoV steel is a mixed mode of intergranular fracture and transgranular fracture, while the failure mode of the novel steel is intergranular fracture. The cyclic softening of the two materials can be attributed to the lath structure with a high density of dislocations gradually transforms into low energy subcrystalline and cellular structures at 700 °C. The novel steel has a better fatigue life than the 30SiMn2MoV steel at 700 °C and different strain amplitudes, which is mainly related to the carbides and lath martensite in the materials.

## 1. Introduction

During the shooting process, the inner bore of the barrel is subjected to repeated effects of high temperature and high pressure, high-speed gas, medium corrosion, etc., in a short period of time. At the same time, it also resists the squeeze and abrasion of the projectile’s reverse side. Therefore, the working conditions are very harsh. The life of gun barrels, which are the core component in guns, remains the limiting factor that affects gun performance. The gun barrel life includes the ballistic life and fatigue life, which are caused by damage to the bore and material fatigue [1]. In actual working conditions, the gun barrel often suffers fatigue failure due to the cyclic load at high temperatures and high pressures, so the fatigue life is very important to gun design [2]. In general, the fatigue life of a gun barrel is not more than 10^5^ cycles, thus, gun barrel fatigue can be regarded as a low cycle fatigue (LCF) behavior under the combined actions of heat and bore pressure. In addition, under the conditions of modern warfare, rapid-fire weapons with high bore pressure, high velocity and long life are increasingly sought. In the process of high-temperature and high-pressure, the gun barrel will experience more cyclic deformation. Therefore, the fatigue reliability of gun barrel is very important.

The novel steel is a new type of Cr–Mo–V gun steel. The LCF behavior of Cr–Mo–V steel has been widely studied by researchers. The low-cycle fatigue research on Cr–Mo–V steel is as follows: Y. Zhang, et al. [3] studied the LCF behavior of a Cr–Mo–V matrix-type high-speed steel at room temperature under strain-controlled cyclic loading conditions with different axial strain amplitudes. The results showed that stress responses at different strain levels exhibited softening behaviors, and the behavior of the alloy is approximated to that of a Masing-type material. Zhiqiang Li, et al. [4] investigated the cyclic stress–strain response and the LCF behavior of a Cr–Mo–V low alloy steel over a range from room temperature to 600 °C. The steel exhibited cyclic softening behavior and behaved as a Masing type, especially at high strain amplitudes. Wenlan Wei, et al. [5] studied the cyclic hardening and dynamic strain aging during low-cycle fatigue of Cr-Mo tempered martensitic steel in the temperature range from 250 to 450 °C, the results showed the cyclic softening of the steel was closely related to the DSA effect, which was manifested by the difference in the dislocation substructure evolution during LCF. Preeti Verma, et al. [6] studied the LCF behavior of modified 9Cr-1Mo steel at 300 °C and at different strain rates. The results showed that the inverse effect of strain rate is observed on LCF behavior. Regardless of strain rate and strain amplitude there is cyclic softening until failure. The cyclic softening is attributed mainly to recovery of dislocations and formation of cell structure. Stanisław Mroziński, et al. [7] investigated the LCF behavior of P91 steel at 20 °C and 600 °C. It is demonstrated that the analytical models using strain-based characteristics of fatigue do not reflect the actual test conditions at σ_a_ = const. The conclusions are confirmed by the analysis of the test results with the use of energy parameters of fatigue. In summary, there are very limited studies on the LCF behavior of Cr–Mo–V steel at 700 °C. Moreover, the literature [8,9] reported that the inner bore surface temperature of the gun barrel can reach approximately 600–700 °C when the gun was shooting. Therefore, it is necessary to study the fatigue performance of gun steels at 700 °C.

At high temperature the strength of most of the present gun steels decrease greatly, especially at 700 °C, which shorten the ballistic life of gun barrels. In order to increase the ballistic life of gun barrels, a novel steel (MPS700V) is optimized to obtain excellent high-temperature strength by adjusting the content of alloying elements such as Cr, Mo and V. As the ballistic life increases, the gun barrel will experience more cyclic loading at high temperature, especially during the continue shooting process, which even leads to the slightly deformation of gun barrels. Therefore, the low cycle fatigue performance of the novel barrel steel is necessary to evaluate the fatigue life in the use of the gun barrel. Compared with the Cr–Mo–V steels mentioned above, the novel gun steel has a relatively lower content of alloying elements, but its high temperature performance is better. This may lead to different high-temperature fatigue behavior mechanisms. Additionally, the LCF behavior of gun steel is still a lack of known. Especially for the newly developed novel steel, the LCF behavior can be different from the traditional 30SiMn2MoV steel due to its higher microstructure thermal stability and high-temperature wear resistance. Therefore, in this paper, LCF behavior was carried out for both the novel steel and 30SiMn2MoV steel, and corresponding failure mechanism was further studied. Furthermore, the fatigue model was proposed to predict the fatigue life of the novel steel, which supports the application for the novel steel.

## 2. Materials and Methods

**Figure 1:**
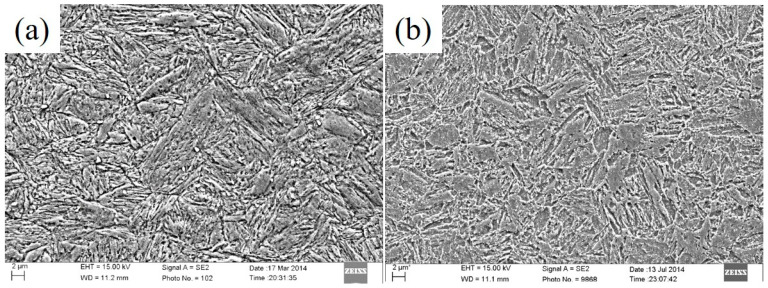
Tempering SEM microstructure of the experiment material: (**a**) novel steel and (**b**) 30SiMn2MoV.

The chemical composition of the novel steel and the 30SiMn2MoV steel (GB/T 3079-93) is given in Table 1. The heat treatment procedure for the novel steel was as follows: it was heated at 1000 °C for 1 h, quenched and tempered at 660 °C for 4 h. The heat treatment procedure for the 30SiMn2MoV was as follows: it was heated at 880 °C for 1 h, quenched and tempered at 620 °C for 4 h. The microstructure of the novel steel and 30SiMn2MoV steel comprised tempered sorbite, as shown in Figure 1.

**Figure 2:**
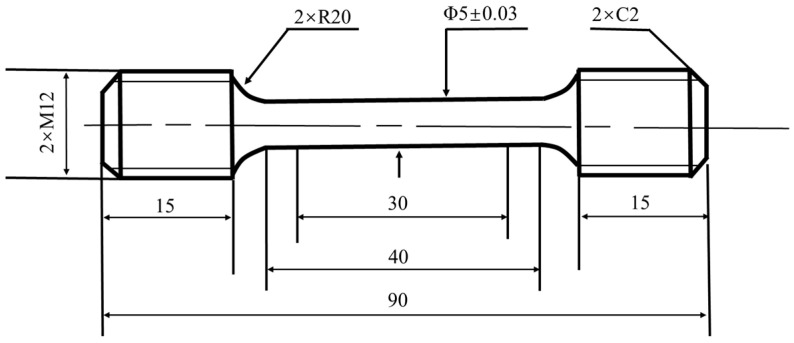
High temperature tensile sample.

According to ASTM E21-2009 “High Temperature Tensile Test Methods of Metallic Materials”, a circular proportional sample was selected for this experiment, as shown in Figure 2. The standard stipulates that the sample is heated to the test temperature within 30 min, and then the test was started after the specified test temperature was maintained for at least 10–15 min.

**Figure 3:**
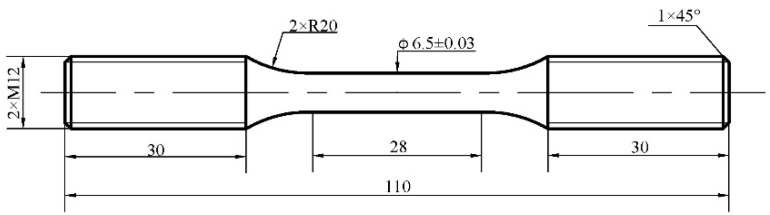
Low cycle fatigue (LCF) test sample.

The test drawing of standard LCF specimens is shown in Figure 3. According to the ASTM E606/E606M-2012 standard, an MTS-810 testing machine was used to perform fatigue tests through a series of fully reversed strain-controlled strain ratios (R = −1) and the strain control waveform was the triangle load move at constant 700 °C, and different total strain amplitudes (0.2%, ±0.4% and ±0.6%) were applied. The strain was controlled with a 25 mm extensometer and the strain rate was controlled at 5 × 10^−3^ × s^−1^. The criterion for the end of a fatigue test was assumed, and the fatigue life N_f_ at a given strain level was the number of cycles at which the occurrence of deformation on the hysteresis loop arm in the compression half-cycle was observed.

The fracture morphology of the sample was observed with a FEI Quanta 250 (Fei Company, Hillsboro, OR, USA) scanning electron microscope. A Tecnai F30 (Fei Company, Hillsboro, OR, USA) field emission transmission electron microscope was used to observe the morphology, dislocation structure, and grain boundaries of carbides. After mechanically thinning the 0.3 mm sheet cut from the sample line near the fracture to 50 μm, it was punched into a 3 mm diameter disc, and then a TEM sample was prepared using an electrolytic dual-spray thinning method.

## 3. Results

### 3.1. High Temperature Tensile Properties

**Figure 4:**
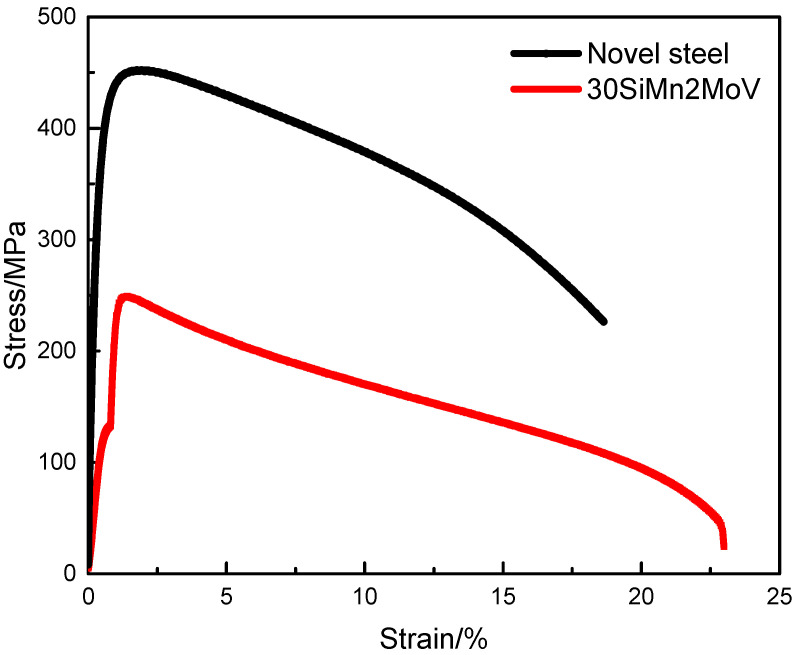
Monotonic tensile stress-strain curves of the novel steel and 30SiMn2MoV steel at 700 °C.

The stress–strain curves of the novel steel and 30SiMn2MoV steel at 700 °C are shown in Figure 4. From Figure 4, it can be seen that the ultimate tensile strength of the novel steel at 700 °C was nearly twice that of 30SiMn2MoV steel, and the yield strength of the novel steel at 700 °C was nearly three times that of 30SiMn2MoV steel, which indicates that the deformation of the novel steel was smaller under the same stress. The mechanical properties of the two steels at 700 °C are summarized in Table 2.

### 3.2. Cyclic Stress Response Behavior

**Figure 5:**
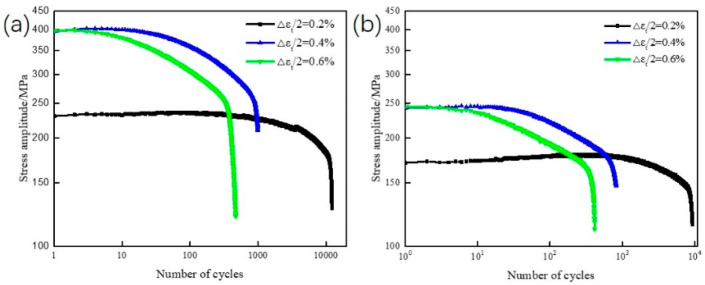
Cyclic stress response of the test steel at 700 °C: (**a**) novel steel and (**b**) 30SiMn2MoV.

The cyclic stress response curve indicates the cyclic hardening or softening behavior of the material under different experimental conditions. The stress amplitude and fatigue life were closely related to the changes in the strain amplitude. The stress response curves of the novel steel and 30SiMn2MoV steel obtained at 700 °C at different strain amplitudes are depicted in Figure 5. An obvious cyclic softening behavior of the novel gun and 30SiMn2MoV steel can be observed and the LCF life of both materials decreased with increasing strain amplitude. The cyclic softening behavior of the two steels in this paper was consistent with that of other gun materials [10].

With an extension in the number of fatigue cycles, the cyclic stress response curve of the novel steel and 30SiMn2MoV steel contains three stages: the early cyclic stability stage, the middle cyclic softening stage and final transient fracture stage. According to the proportion of three different stages, the proportion of cyclic stability stage in the fatigue life was less than 2%, the proportion of cyclic softening stage in the fatigue life was more than 90% and the final instantaneous fracture stage was due to the instantaneous failure of the material and rapid decrease in the stress. When the total strain amplitude was 0.2%, 0.4% and 0.6%, the initial cyclic stresses of the novel steel were 230 MPa, 395 MPa and 397.5 MPa respectively; those for the 30SiMn2MoV steel were 170.5 MPa, 242 MPa and 244.5 MPa respectively. Compared with that for the 30SiMn2MoV steel, the stress of the novel steel at the same strain amplitude was higher, which was consistent with the trend for the tensile stress and strain at 700 °C. Upon increasing the strain amplitude from 0.2% to 0.6%, the fatigue cyclic stress and the cyclic softening rate of the tested materials increase, and the number of fatigue cycles in the stable cycle decreased gradually.

### 3.3. Cyclic Stress–Strain Behavior

**Figure 6:**
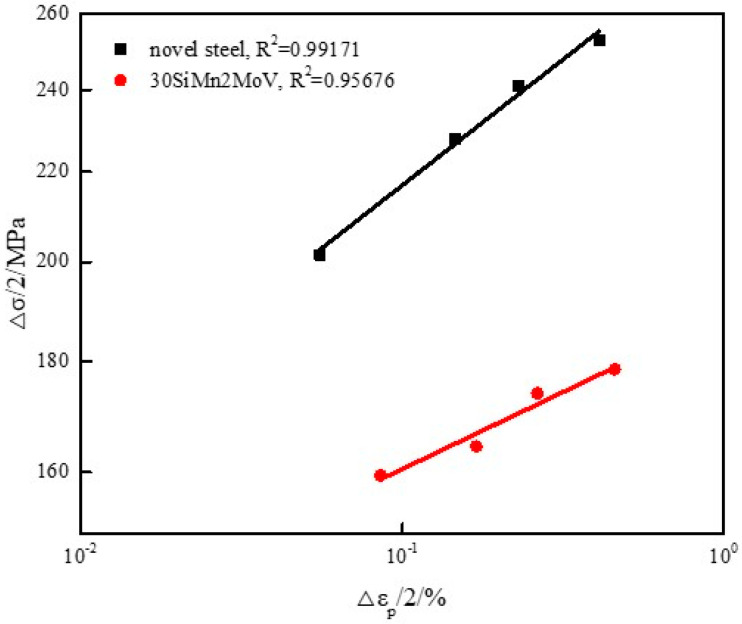
Cyclic stress amplitude versus plastic strain amplitude of the novel steel and 30SiMn2MoV steel at 700 °C.

The cyclic stress–strain behavior reflects the true stress–strain characteristics of materials under low-cycle fatigue conditions, which is an important aspect of low-cycle fatigue research. The relationship between the cyclic stress amplitude and the plastic strain amplitude of the novel steel and 30SiMn2MoV steel at 700 °C is shown in Figure 6. The data points of the Figure 6 were obtained from the cyclic hysteresis loop at half-life. The cyclic stress–strain curve of materials can be expressed in the following Equation (1) [11]:

(1) $$\Delta \sigma /2=K^{\prime }{\left(\Delta \varepsilon_{p}/2\right)}^{n^{\prime }}$$

where, Δσ/2 is the cyclic stress amplitude, $\Delta \varepsilon_{p}/2$ is the plastic strain amplitude, $K^{\prime }$ is the cyclic strength coefficient and $n^{\prime }$ is the cyclic strain hardening index. According to Equation (1), linear regression analysis was performed using double logarithmic coordinates,$\;K^{\prime }$, $n^{\prime }$ and the relationship between cyclic stress amplitude and plastic strain amplitude of the novel steel and 30SiMn2MoV steel at 700 °C are shown in Table 3. Table 3 shows that the cyclic stress–strain parameters ($K^{\prime }$ and $n^{\prime }$) of the novel steel were all larger than those of the 30SiMn2MoV steel, which indicates that the plastic deformation of the novel steel could be reduced when the barrel was subjected to the same applied stress during shooting, thus the shooting accuracy of the barrel could be improved.

### 3.4. Masing Analysis

**Figure 7:**
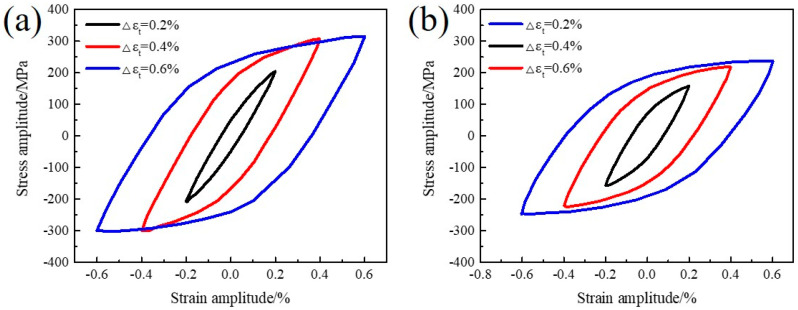
The superimposed hysteresis loops of the specimens at different strain levels: (**a**) novel steel and (**b**) 30SiMn2MoV.

The area of the hysteresis ring represents the energy consumed by external forces during the plastic deformation of the material, and also represents the ability of the material to resist cyclic deformation, which is also called cyclic toughness. Figure 7 presents the half-life cyclic stress–strain hysteresis loops with different strain amplitudes for the novel steel and 30SiMn2MoV steel. Figure 7 shows that the hysteresis loop area of the novel steel and 30SiMn2MoV steel increased with increasing strain amplitude, which shows that the cyclic toughness of the two materials increased gradually. In the same strain range, the novel steel had higher resistance to plastic deformation because the greater stress range led to a larger area of the hysteresis curve.

Figure 7 shows that the area of the hysteresis loop increased with increasing total strain amplitude, namely, the accumulation of plastic deformation during each cycle also gradually increased with increasing strain amplitude. The upper branches of a stable half-life hysteresis loops with different strain amplitudes (Figure 7) were moved to the same location as the lowest points of each hysteresis loop for a material with Masing characteristics. Under cyclic loading with different strain amplitudes, Masing materials show the same proportional limit, so the upper half of their hysteresis loops can overlap. For a non-Masing material, the performance of the proportional limits is different as the strain amplitudes are different, so the hysteresis loops cannot overlap.

**Figure 8:**
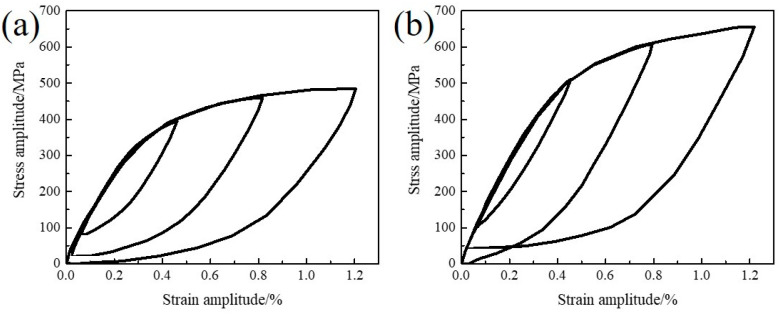
The superimposed hysteresis loops along the linear portion to match upper branches (**a**) novel steel and (**b**) 30SiMn2MoV.

The saturated curves of the novel steel and 30SiMn2MoV steel are shown in Figure 8. It is obvious that the ascending branches at different strain amplitudes did not follow a unique curve, and neither material exhibited Masing-type behavior; therefore, these were non-Masing materials.

### 3.5. Low Cycle Fatigue Life

The fatigue test results of the novel gun and 30SiMn2MoV steel at 700 °C are shown in Table 4. The values of $\Delta \varepsilon_{e}/2$ and $\Delta \varepsilon_{p}/2$ were obtained from the half-life stress-strain hysteresis loops.

Based on the well-known Basquin [12] and Manson [13]–Coffin [14] formulations, the elastic strain amplitude, plastic strain amplitude and the number of reversals to failure have an exponential relationship and can be expressed by Equations (2)–(4):

(2) $$\frac{\Delta \varepsilon_{t}}{2}=\frac{\Delta \varepsilon_{e}}{2}+\frac{\Delta \varepsilon_{p}}{2}=\frac{\sigma_{f}{}^{\prime }}{E}{\left(2N_{f}\right)}^{b}+\varepsilon_{f}{}^{\prime }{\left(2N_{f}\right)}^{c}$$

(3) $$\frac{\Delta \varepsilon_{e}}{2}=\;\frac{\sigma_{f}{}^{\prime }}{E}{\left(2N_{f}\right)}^{b}$$

(4) $$\frac{\Delta \varepsilon_{p}}{2}=\varepsilon_{f}{}^{\prime }{\left(2N_{f}\right)}^{c}$$

where $\Delta \varepsilon_{t}/2$ is the total strain amplitude; $\sigma_{f}{}^{\prime }$ is the fatigue strength coefficient, MPa; E is the elastic modulus of the test material at 700 °C, MPa; b is the fatigue strength index; $\varepsilon_{f}{}^{\prime }$ is the fatigue ductility coefficient and c is the fatigue ductility index. $\Delta \varepsilon_{t}$, $\Delta \varepsilon_{e}$ and $\Delta \varepsilon_{p}$ are all from the half-life hysteresis cycle. $\sigma_{f}{}^{\prime }$ and b describe the mechanical properties of the material during the elastic deformation stage of the fatigue test and the effect on the fatigue life of the material. $\varepsilon_{f}{}^{\prime }$ and c describe the influence of the plastic deformation stage on the fatigue life of the material.

**Figure 9:**
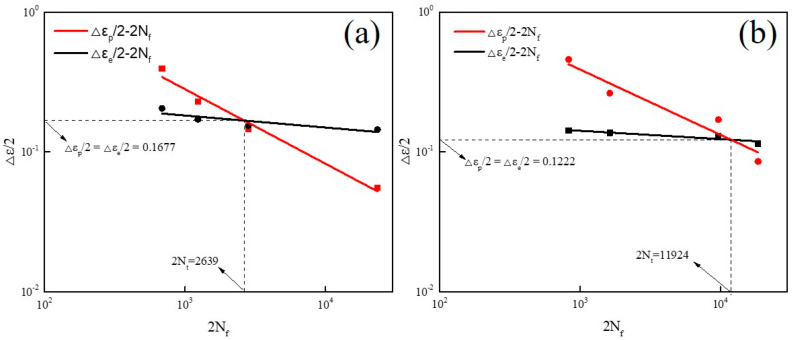
Manson–Coffin curves of test materials: (**a**) novel steel and (**b**) 30SiMn2MoV.

According to Equations (3) and (4), a double logarithmic linear regression analysis is performed on Figure 9 to obtain the fatigue parameters of the novel gun and 30SiMn2MoV steel, which are shown in Table 5. By substituting each value into Equation (2), the relationship between the plastic strain amplitude and the number of reversals to failure was obtained. According to this equation, the LCF life of the novel steel and 30SiMn2MoV steel at 700 °C could be predicted. The fatigue ductility index c is the slope of the plastic strain–LCF life curve and the fatigue strength exponent b is the slope of the elastic strain–LCF life curve. The higher the absolute values of parameters c and b, the less the fatigue life decreased at a given increase in plastic strain and elastic strain. From Table 5, the absolute values of parameter c and b of the novel steel were higher, which indicates that the novel steel had a higher fatigue ductility.

Generally, when the plastic strain amplitude is exactly equal to the elastic strain amplitude (the intersection of the two fitted lines of $\Delta \varepsilon_{e}/2-2N_{f}$ and $\Delta \varepsilon_{p}/2-2N_{f}$ in Figure 9), the fatigue life at the intersection is called the transition life $N_{t}$ and $N_{t}$ is considered to be a key indicator of the LCF performance of materials [15]. The factors that affect the transient fatigue life are mainly the strength and plasticity of the material [16]. The higher the strength of a material and the lower the plasticity are, the lower its transition fatigue life. When $N_{f}\;$<$\;N_{t}$, the plastic strain is greater than the elastic strain, the plastic strain contributes more to the fatigue than the elastic strain, and the plasticity of the material has a major role in the fatigue resistance. When $N_{t}\;$<$\;N_{f}$, the elastic strain is greater than the plastic strain, and the effect of the elastic strain on the fatigue life is greater than that of the plastic strain, that is, the strength of the material plays a major role in the fatigue resistance. Figure 7 shows that the $N_{t}$ of the novel steel was 1318 cycles, and that of 30SiMn2MoV was 5962 cycles. The corresponding critical total strain amplitudes of the novel steel and 30SiMn2MoV steel were 0.3354% and 0.2444%, respectively.

### 3.6. Energy–Life Prediction Models

The theory of plastic strain energy holds that cyclic deformation and its accumulation are the basic causes of fatigue damage, and the plastic strain energy density (${\Delta w}_{p}$) is an important parameter to describe the fatigue damage. The cyclic hysteresis energy refers to the irreversible plastic work consumed per cycle during the cyclic process, and it is equal to the area enclosed by the corresponding cyclic hysteresis loop.

The relation between the plastic strain energy and fatigue life mentioned above can be described by the Halford–Marrow Equation (5) [17].

(5) $${\Delta W}_{p}=w_{f}^{\prime }{\left(2N_{f}\right)}^{\beta }$$

where 2$N_{f}$ is the number of reverse cycle failures under each strain; $w_{f}^{\prime }$ is the fatigue plastic strain energy parameter and β is the fatigue plastic energy index. All the values of these parameters are listed in Table 6. For non-Masing materials, the plastic strain energy can be calculated from the following equation [18]:

(6) $${\Delta w}_{p}=\frac{1-n^{*}}{1+n^{*}}\Delta \sigma \Delta \varepsilon_{p}+\frac{2n^{*}}{1+n^{*}}\delta \Delta \sigma_{0}\Delta \varepsilon_{p}$$

where n* is the hardening exponent of the master curve and $\Delta \sigma_{0}=\Delta \sigma -\Delta \sigma^{*}$. The master curve is a unique curve that defines the matched hysteresis loop branches at different strain amplitudes.

**Figure 10:**
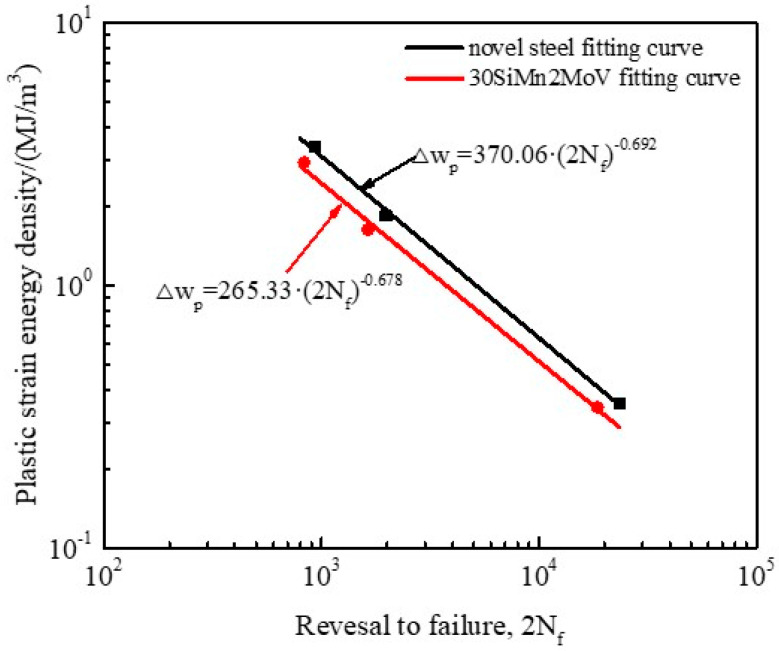
Comparation of plastic strain energy density ${\Delta w}_{p}$ between the novel steel and 30SiMn2MoV steel.

The data calculated by Equation (1) at different strain amplitudes and temperatures are plotted in Figure 10. The results show that the plastic strain energy density of the novel steel and 30SiMn2MoV steel decreased with increasing fatigue life. In other words, increasing the total strain range could significantly increase the plastic strain energy density of the two materials at 700 °C. The experimental data were in good agreement with the fitting curve. The slope (β) and intercept ($w_{f}^{\prime }$) of the ${\Delta w}_{p}$-2N_f_ curve are shown in Table 6. From Table 6, the $w_{f}^{\prime }$ value of the novel steel was approximately 1.4 times that of the 30SiMn2MoV steel, which means that the plastic strain energy absorbed by the novel steel sample at 700 °C was 1.4 times higher than that of the 30SiMn2MoV steel sample when it was broken in a single cycle. From the results in Figure 10, the fatigue life of the novel steel was longer than that of 30SiMn2MoV steel under the same plastic strain energy density values. This further illustrates that the novel steel had better fatigue behavior than the 30SiMn2MoV steel.

**Figure 11:**
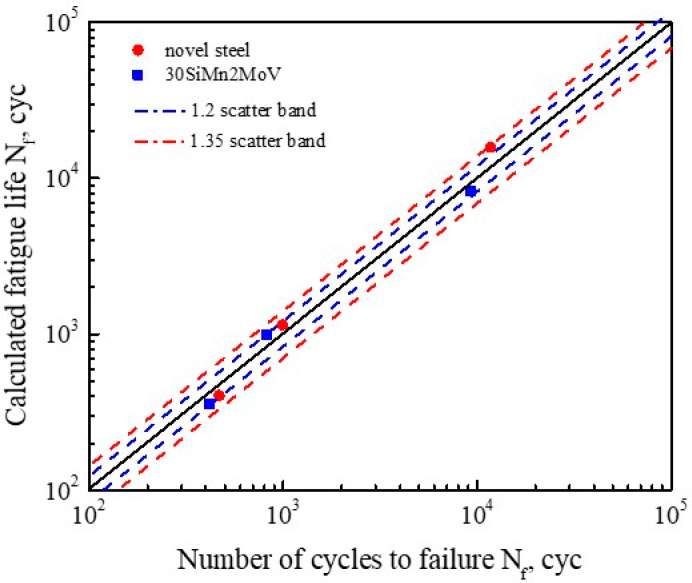
The comparison of calculated values of fatigue life with experimental life.

To verify the reliability of the model, we plotted the calculated fatigue life curve and the test fatigue life curve as shown in Figure 11. The experimental data of the novel steel and 30SiMn2MoV steel were included within a 1.35 and 1.2 scatter band of the prediction, respectively. This implies that the energy–life relationship predicted the fatigue life behavior well for the novel steel and 30SiMn2MoV steel.

### 3.7. Fatigue Fracture Morphology

**Figure 12:**
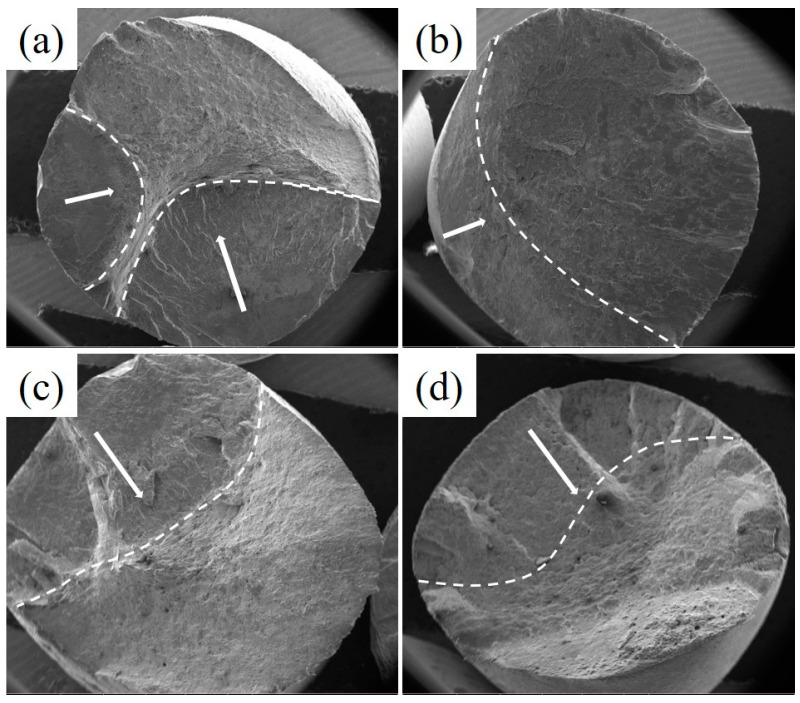
Overall morphologies of two materials, novel steel: (**a**) 0.2% and (**b**) 0.6%, and 30SiMn2MoV: (**c**) 0.2% and (**d**) 0.6%.

The fracture morphologies of the LCF samples with different strain amplitudes of 0.2% and 0.6% at 700 °C are exhibited in Figure 12. Figure 12 shows the typical characteristics with the three regions comprising the fatigue source, fatigue crack propagation zone and final fracture zone [19]. It can be seen from Figure 12 that when the strain amplitude increased from 0.2% to 0.6%, the crack propagation area decreased, the number of crack initiation sources of the novel steel was less than that of the 30SiMn2MoV steel and the crack propagation area was greater than that of 30SiMn2MoV steel with the same strain amplitude. In general, the decrease in the crack propagation area indicates a decrease in the fatigue life, which was consistent with the fatigue life results in Table 3.

**Figure 13:**
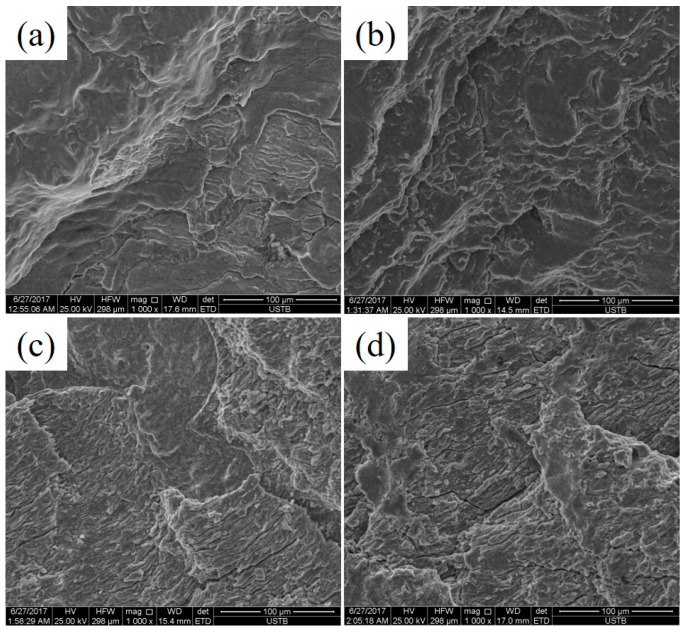
Typical morphologies of propagation zone at different strain amplitudes, novel steel: (**a**) 0.2% and (**b**) 0.6%, and 30SiMn2MoV: (**c**) 0.2% and (**d**) 0.6%.

The fatigue crack propagation zones of the novel steel and 30SiMn2MoV steel under different strain amplitudes are shown in Figure 13. Fatigue striation can be observed in Figure 13. The fatigue striations reflected the ductility of the material and the process of fatigue crack propagation, which was influenced by the chemical composition, microstructure and cyclic loading. The fatigue striation interval indicates the propagation of cracks during a loading cycle, and the crack propagation direction was perpendicular to the direction parallel to the fatigue striations [20]. Compared to the fracture surface of the two tested materials, the fatigue striation characteristics of the 30SiMn2MoV steel were more obvious, and intergranular cracks existed in the fatigue crack propagation zone. However, intergranular cracks were not seen in the fatigue crack propagation region of the novel steel, which indicates that the intergranular fracture mode of 30SiMn2MoV steel played an important role during failure. Therefore, the failure mode of the 30SiMn2MoV steel was a mixed mode of intergranular fracture and transgranular fracture, however the failure mode of the novel steel was the intergranular fracture.

### 3.8. TEM Observation of Fatigue Microstructure

**Figure 14:**
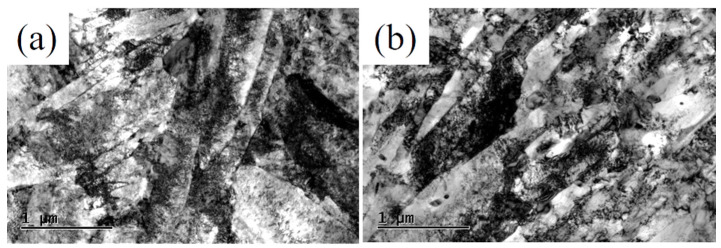
TEM microstructures of tempered samples: (**a**) novel steel and (**b**) 30SiMn2MoV.

**Figure 15:**
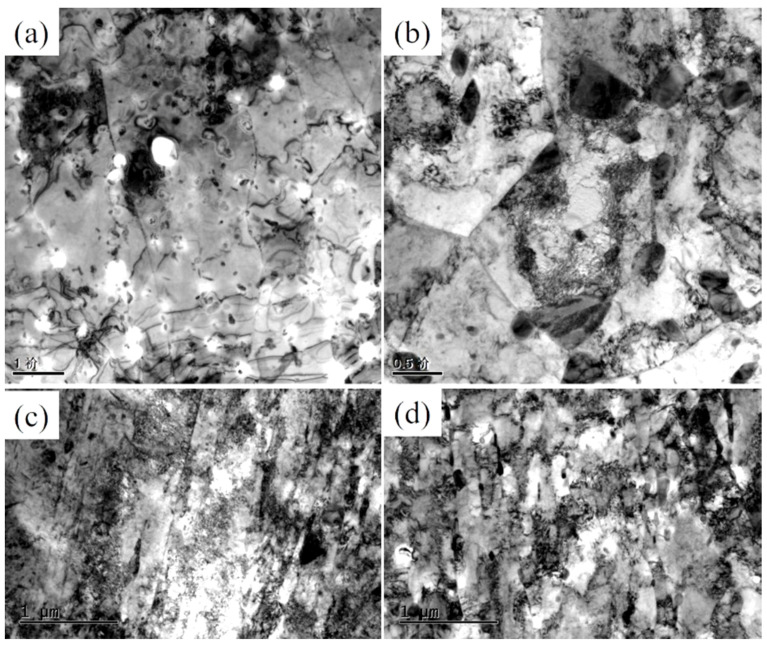
TEM microstructures of both steels at different strains. (**a**) 0.2%, Novel steel; (**b**) 0.2%, 30SiMn2MoV steel; (**c**) 0.6%, Novel steel; (**d**) 0.6%, 30SiMn2MoV steel.

The quenched-tempered TEM microstructures of the novel steel and 30SiMn2MoV steel are shown in Figure 14. From Figure 14, it can be concluded that the microstructures of the novel steel and 30SiMn2MoV steel both show lath-shaped martensite structures, high densities of dislocations and large amounts of carbides in and within the laths. The TEM microstructures of the novel steel and 30SiMn2MoV steel at strain amplitudes of 0.2% and 0.6% are shown in Figure 15. It shows that the lath structure with high-density dislocations in the novel steel and 30SiMn2MoV steel gradually transforms into low-energy subcrystals and cell structures, indicating that dynamic recovery and dynamic recrystallization occurs during the high temperature LCF.

At a strain amplitude of 0.2%, the tempered martensite laths of the two steels almost disappeared and transformed into cell structures of low-density dislocations. There were many pinned dislocations and carbides in the subgrains and grain boundaries (Figure 15a,b). However, the size of the carbides in the novel steel ranged from approximately 50 to 100 nm, and the size of carbides in 30SiMn2MoV steel ranged from approximately 200 to 500 nm. It is obvious that the carbides in the 30SiMn2MoV steel were much larger than those in the novel steel.

At a strain amplitude of 0.6%, the structure of the novel steel after low-cycle fatigue at 700 °C observed with TEM still shows lath characteristics, while the 30SiMn2MoV steel was recrystallized. Compared with the microstructure before the test, the number of carbides in the matrix of the two materials did not change significantly (Figure 15c,d).

## 4. Discussion

The tensile strength and yield strength of the novel steel at 700 °C were much higher than those of 30SiMn2MoV steel (Figure 4). The reason may be that the novel steel contains more Mo and Cr that form M_2_C and M_23_C_6_ phases [21,22]. In the process of high-temperature elongation, the carbides of M_2_C and M_23_C_6_ are distributed in the martensite matrix, pinning dislocation movement and hindering dislocation movement, thus improving the high-temperature strength of materials [22]. The true stress–strain curves of both steels all show cyclic softening. Nevertheless, the novel steel has a higher cyclic strength than the 30SiMn2MoV steel (Figure 5), which is consistent with the tensile test results. The reason for cyclic softening is that the dislocation structure of the material begins to rearrange, defects, such as high-density vacancies and dislocations, are also recovered, and the recrystallization and lath structure of the matrix material are changed into a cellular structure (Figure 14 and Figure 15). These observations were consistent with those in earlier studies.

The fatigue life of the two materials decreased as the strain amplitude increased (Figure 5), and the fatigue life of the novel steel was significantly higher than that of 30SiMn2MoV steel in the same strain amplitude (Table 4). The fatigue life decreased with increasing strain amplitude, possibly because there was an increase in the plastic strain amplitude caused by the accumulation of fatigue damage during each cycle [23]. Under the same strain amplitude, the plastic strain amplitude of the novel steel was lower than that of the 30SiMn2MoV steel (Table 4), which also confirms that the novel steel had an improved cycle toughness (Figure 7). In addition, by analyzing the fracture morphology of the two materials after LCF, it can be seen that the number of crack initiation sources in the novel steel was less than that in the 30SiMn2MoV steel, and the crack growth area of the novel steel was greater than that in the 30SiMn2MoV steel, and there were fewer secondary cracks in the crack growth area of the novel steel. The failure mode of the 30SiMn2MoV steel was a mixed mode of intergranular fracture and transgranular fracture; however, the failure mode of the novel steel was intergranular fracture. The fracture path of transgranular cracks was short, which reduced the fatigue resistance of the base metal. Therefore, the novel steel had better fatigue ductility and a longer fatigue life than the 30SiMn2MoV steel. As the high-temperature strength of 30SiMn2MoV steel was lower than that of the novel steel at 700 °C, the grain boundary strength of 30SiMn2MoV steel was weaker and easily led to transgranular fracture.

The novel steel had a higher fatigue life under different strain amplitudes. However, the mechanisms of the different strain amplitudes were different. When the strain amplitude was 0.2%, the volume and quantity of the carbides played a major role in the fatigue failure. The lath structure in both materials recrystallized to form cellular and subcrystalline structures. However, the carbides in the 30SiMn2MoV steel were coarsened, and the carbide volume was much larger than that of the novel steel (Figure 15a,b). The large volume of carbides caused dislocations to accumulate and entangle, which could easily lead to stress and crack initiation (Figure 12a,c). Dislocation pinning and entanglement occurring around the large volume carbide led to stress concentration, so the 30SiMn2MoV steel was more likely to reach the critical size of the crack and initiated microcracks (Figure 13), and the fatigue life of the 30SiMn2MoV steel was shorter. When the strain amplitude was 0.6%, martensite features played a major role in fatigue failure. Since the number of cycles was small and the holding time was short, precipitation and coarsening of the carbides did not have sufficient time to occur. However, the novel steel still retained the lath martensite, while the 30SiMn2MoV steel underwent recrystallization. The tempered lath martensite had an increased dislocation density [24], strength and hardness. The laths were interphased at a small angle between the grain boundaries, and the phase difference is small, so it is more difficult to slip between the martensite laths. Moreover, it is also difficult for cracks to pass through the lath martensite bundles from initiation to growth, which leads to a high fatigue limit of the material [25].

## 5. Conclusions

1. The novel steel and 30SiMn2MoV steel all show cyclic softening during the LCF testing at 700 °C. The cyclic stress–strain equation and strain fatigue life equation of both steels at 700 °C were obtained in this paper.
2. The fatigue life of the two steels decreased obviously with strain amplitude increasing from 0.2% to 0.6%, and the novel steel had a higher fatigue life under the same strain amplitude. According to the hysteresis loops under different strain amplitudes, both steels demonstrated non-Masing behavior. The prediction of the fatigue life was in good agreement with the plastic strain energy density model, and the novel steel had better fatigue resistance than the 30SiMn2MoV steel.
3. Fatigue cracks in the novel steel and 30SiMn2MoV steel predominantly initiate on the surface of the samples. The failure mode of the 30SiMn2MoV steel was a mixed mode of intergranular fracture and transgranular fracture; however, the failure mode of the novel steel was intergranular fracture.
4. The novel steel had better fatigue performance at 700 °C. At low strain amplitude and high strain amplitude, the fatigue performance depended on the carbide morphology and martensitic lath characteristics in the material, respectively.

## Figures and Tables

**Table 1:** Comparison of the chemical composition between the novel steel and 30SiMn2MoV steel.

Steel	C (%)	Si (%)	Mn (%)	Cr (%)	Mo (%)	W (%)	Ni (%)	V (%)
Novel Steel	0.25- 0.28	–	–	2.50- 2.80	1.60- 1.90	0.20- 0.60	0.50- 0.80	0.20- 0.50
30SiMn2MoV	0.27- 0.32	0.40- 0.60	1.60- 1.85	0.10- 0.25	0.40- 0.60	–	0.10- 0.25	0.15- 0.25

**Table 2:** The mechanical properties of the novel steel and 30SiMn2MoV steel at 700 °C.

Steel	Tensile Strength (MPa)	Yield Strength (MPa)	Elongation (%)	Reduction (%)
Novel Steel	452	350	30	82.4
30SiMn2MoV	240	119	42	82

**Table 3:** The cyclic stress–strain parameters of the novel steel and 30SiMn2MoV steel.

Material	$K^{\prime }$	$n^{\prime }$
Novel Steel	283.23	0.1158
30SiMn2MoV	189.08	0.0709

**Table 4:** LCF test results of the novel steel and 30SiMn2MoV steel.

Material	$\Delta \varepsilon_{t}/2(\%)$	$\Delta \varepsilon_{e}/2(\%)$	$\Delta \varepsilon_{p}/2(\%)$	$2N_{f}$
Novel Steel	0.2	0.14465	0.0555	23,462
	0.4	0.17125	0.229	1980
	0.6	0.19075	0.409	936
30SiMn2MoV	0.2	0.1146	0.0855	18,604
	0.4	0.1349	0.265	1634
	0.6	0.14225	0.4575	832

Note: that $\Delta \varepsilon_{t}/2$ is the total strain amplitude, $\Delta \varepsilon_{e}/2$ is the elastic strain amplitude, $\Delta \varepsilon_{p}/2$ is the plastic strain amplitude, 2$N_{f}$ is the number of f reversals to failure.

**Table 5:** LCF parameters of different materials.

Material	$\sigma_{f}{}^{\prime }/E$	$\varepsilon_{f}{}^{\prime }$	b	c
Novel Steel	0.3299	11.12	−0.0859	−0.5323
30SiMn2MoV	0.2134	9.7629	−0.0594	−0.4669

**Table 6:** Energy-based properties of the novel steel and 30SiMn2MoV steel.

Material	$w_{f}^{\prime }$	β
Novel Steel	370.06	−0.692
30SiMn2MoV	265.33	−0.678
